# Supplementary material for: Artificial intelligence-designed single molar dental prostheses: A protocol of prospective experimental study
Source: PLoS One. 2022 Jun 2;17(6):e0268535. doi: 10.1371/journal.pone.0268535 (PMC9162350; doi:10.1371/journal.pone.0268535)
Supplement: S1 File — (DOCX) [file pone.0268535.s002.docx]

Fig 1 [SPIRIT Checklist] was used under the terms of the Creative Commons “Attribution-NonCommercial-NoDerivs 3.0 Unported” license.

Fig 2 [Brief architecture of the Generative Adversarial Network (GAN)] was created by author Reinhard Chun Wang Chau

Fig 3 [General flow of processing collected data] was created by author Reinhard Chun Wang Chau

Fig 4 [System specification of High-Performance Computer] was published under permission by the copyright holder with proof uploaded.

Fig 5A [Illustration of voxel-based method] was created by author Reinhard Chun Wang Chau

Fig 5B [Illustration of view-based method] was created by author Reinhard Chun Wang Chau

Fig 5C [Illustration of point-based method] was created by author Reinhard Chun Wang Chau

Fig 6A&6B [Example of a maxillary teeth model and a mandibular teeth model] was created by author Reinhard Chun Wang Chau

Fig 6C [Demonstration of maxillary teeth model and its antagonist mandibular teeth model at maximal intercuspal position] was created by author Reinhard Chun Wang Chau

Fig 7A&7B [Superimposition of teeth for comparison. Measurement of the geometric morphology and 3D position by locating the anatomical landmarks of a tooth such as cusp tips and fossae as well as the center of a tooth (arrowed) respectively.] was created by authors Ming Chong and Walter Yu Hang Lam
