# Supplementary material for: Artificial intelligence-designed single molar dental prostheses: A protocol of prospective experimental study
Source: PLoS One. 2022 Jun 2;17(6):e0268535. doi: 10.1371/journal.pone.0268535 (PMC9162350; doi:10.1371/journal.pone.0268535)
Supplement: S1 Protocol — (PDF) [file pone.0268535.s004.pdf]

Title: The use of AI in predicting 3D position and occlusal morphology of single missing teeth

Version 1.0

Date: 1/12/2020

[Amendments: Not applicable at this initial version]

Introduction

Tooth loss and its consequences

Tooth loss is common among the population and as consequence, patient's health and quality-of-life are deteriorated. Tooth loss is common among the local community particularly the elderly (1, 2). The prevalence of tooth loss is expected to be increased with the ageing population. In the *Hong Kong Population Projectio*n, 25% of the population will be over the age of 60 (3). Tooth loss is anticipated to be a significant problem in the near future. Tooth loss is associated with impaired mastication and nutrition intake (4). Moreover, the loss of function (mastication and speech) and compromised aesthetics are associated with impaired quality of life (5-7).

Dental prostheses and its requirement

Dental prostheses aim to restore patients' appearance and functions by replacement of missing teeth. Loss of single tooth is the most common pattern of tooth loss nowadays (14). Resin-bonded fixed dental prostheses and single-tooth implants are commonly used to replace single missing teeth (8-14), to restore the function as well as the quality of life (15).

The morphology and 3D position of the healthy natural teeth should be adopted by the dental prostheses as so called **biomimetric** (16, 17). As result of evolution, healthy natural teeth have attained the best mechanical and biological properties. Their (occlusal) morphology and 3D position reach the functional equilibrium, i.e. any inharmony will be presented in tooth fracture, increased mobility and/or tooth drifting movement. Functionally, the occlusal (biting) surface of teeth should fit to the opposing (antagonist) teeth in both static and dynamic occlusion (18). The basic concept of occlusion is that the occlusal design of dental prosthesis should correspond to the natural tooth shape in harmonic relationship with the adjacent and antagonistic elements to allow smooth jaw movements (19). The occlusal surfaces are functionally critical: tooth with **over-contoured** occlusal surfaces will hinder jaw movement, receive all or most of the functional loading and will fail easily, while the **under-contoured** occlusal surfaces may result in loss of function and overloading of adjacent teeth. Moreover, the aesthetic of the dental prostheses should be in harmony to the adjacent teeth and the overall appearance of a patient (20).

Reconstructing the occlusal morphology of dental prostheses is a challenge in dentistry (21). Dentists and dental technicians spend significant amount of time to capture patients' information to simulate the jaw movement in the form of facebow and jaw record. This information will transfer to an articulator which is a mechanical device that simulate patients' jaw movement, for fabrication of the occlusal surfaces of dental prostheses. Moreover, the restoration of missing teeth with correct morphology and position requires special attention to the details of the dental (teeth and gum) and facial aesthetics.

Traditionally, dental prostheses are fabricated by dental technicians on stone teeth models that mounted in the articulator. However, the setting of dental stone, waxing up the pattern of dental prostheses and subsequent casting into metal prostheses using lost-wax techniques are all time consuming and the wax patterns are prone to distortion. Moreover, the hand carving of occlusal morphology is technique-sensitive and may be limited by technician's experience.

Current state-of-the-art: Computer-assisted design CAD and virtual patient model VPM

Digital dentistry is the use of computer technology to assist and improve the practice of dentistry by providing more patient information and/or reduce clinical and operation time. In the *first phase* of digital dentistry, imaging equipment such as intraoral scanners (22, 23), photogrammetry (24) and cone beam computed tomography have been used to digitize patients. By combining these imaging (25-27) and the generation of virtual patient models (VPMs), patients' jaw movement and teeth-facial relationship can then be simulated virtually. VPMs provides more information to dentists and dental technicians for precise design of dental prostheses that with better function and aesthetics replacement. Computer have been used to assist the design (computer-assisted design CAD) and manufacturing (computer-assisted manufacturing CAM) of dental prostheses.

Computer-assisted design software facilitate the design of dental prostheses by simulation of patient's jaw movements with increase accuracy (28, 29) and reduction of operative time (30). Virtual articulator, a software program, allow more individualized customization for jaw movements than a mechanical articulator to account for individual factors such as the inter-condylar distance and Bennett movement etc. Virtual patient model (VPM) is one of the recent developments in the digital dentistry. This VPM provide more sophisticated and accurate simulation of patient's jaw movements with the incorporation of patients' 3D face for aesthetics (31-34). The virtual simulation provides more freedom for customization to individual patients than the mechanical articulators.

Current computer-assisted design of dental prostheses and its limitation

Standard libraries have been used in the design of the occlusal morphology of the dental prostheses by the current CAD software. Several tooth morphology styles such as the *biogeneric* tooth model are currently available. However, these built-in styles do not consider the tooth morphology of remaining teeth and usually do not fit to an individual patient. Therefore, considerable input from the dentists and dental technicians are still required (35, 36). A good knowledge of CAD software is a perquisite (37, 38) and considerable clinical time are spent to capture patient's data to program the virtual articulator so as to simulate jaw movement as well as to optimize aesthetics. Lengthy chairside adjustments, which may result in considerable alterations in the tooth morphology, and may compromise the strength and aesthetic of dental prostheses due to altered material thickness (39, 40).

The **duplication** function of the CAD software enables dentists to transfer the occlusal morphology of a tooth pre-operatively to its replacement dental prostheses (41-43). However, this function may have limited application: 1) since there is difficulties in reproducing the tooth morphology in case when the tooth loss is due to severe caries and/or fractures and in many cases, the tooth may be lost when patients visiting restorative dentists. 2) This duplication function enables dentists to transfer the occlusal morphology from the contralateral tooth. However, the long axis and 3D position of duplicated tooth do not follow the natural tooth. The occlusal morphology of the duplicated tooth still requires extensive modification by the dental technicians and by the virtual articulator to fit into patient's mouth.

Future trend and the possible solution

The *second phase* of the digital dentistry is the **automated** dentistry which includes the use of **artificial intelligence** (44) to provide automatic disease diagnosis and clinical decisions as well as the development of **dental robotics** (45-47) for surgery and tooth preparation. Dentistry will move from human-dominant, with and without computer-assisting, to computer- and robotic-dominant. This will reduce the clinical time and therefore reduce the labor cost for the dental treatments. Moreover, the reproducibility and quality of theses **automated** treatments will be improved by elimination of human variations, fatigue and errors.

Artificial intelligence (AI) perform tasks normally requiring human intelligence and has already been commonly used in dentistry for diagnose of diseases in the imaging such as 2D radiography and 3D

computed tomography (48, 49). We can train the AI system to learn how to make clinical decision by deep learning. Different to the traditional computer programming, we do not need to instruct the computer how to make clinical decisions. We only need to instruct the computer to learn the data by defining layers of neural network, the computer will learn the features by its own and then able to make decision. **Our group has trained an AI system to detect the presence of gum inflammation (gingivitis) in the 2D photography** (50).

Our uniqueness and novelty

AI has been used to determine the occlusal morphology and 3D position of teeth that are generated by dental technicians (51, 52). However, the morphology and 3D position of human-determined teeth are prone to error. Our clinical experience suggests the crown produced by dental technicians require considerable intraoral adjustment. The best articulator is the patients' jaw and any current jaw simulation is susceptible to error.

We propose to input the healthy natural teeth for computer to learn the occlusal morphology and 3D position of missing teeth. Teeth of an individual patient are genetically controlled and exposed to almost identical environmental factors in the mouth, therefore the occlusal morphology and 3D position of teeth in a dental arch are inter-related. The cuspal angle of the occlusal surfaces, the tooth morphology (taper, ovoid and square in shape as well as the size of teeth) and the arch size are controlled by gene (53). During the dynamic excursive jaw movements, the contact of teeth between the upper and lower jaw should be arrange to allow smooth and unhindered jaw movements. Any tooth contact that prevents the smooth jaw movement is known as *interference*. Teeth in one side are arranged in canine guidance (i.e. canine only) or group function (i.e. multiple teeth) during excursions and therefore share similar features of occlusal morphology. Similarly, the tooth position is controlled by the force between the tongue and the cheek (neutral zone), the size of teeth and jaw bone, as well as the any premature loss of tooth and subsequent tooth movement. Healthy natural teeth are therefore the best representation of occlusal morphology and 3D position of dental prostheses.

Hypothesis

It is hypothesized that artificial intelligence (AI) can automated designing the single-tooth dental prostheses from the features of remaining dentition. The occlusal morphology and 3D position of the AI-generated tooth is comparable to that of human-designed dental prostheses, when

Aim and Objectives

The overarching aim of this project is to determine whether Artificial Intelligence (AI) is better at predicting the occlusal morphology and 3D position of missing tooth/teeth based on the features of remaining dentition than conventional non-AI approaches.

Objectives:

1. To compare four deep learning methods/algorithms in interpreting and learning the features of 3D models
2. To compare the AI system with maxillary tooth model alone to maxillary and mandibular (antagonist) models
3. a) To compare the occlusal morphology and 3D position of the single-tooth dental prostheses designed by trained AI and by dental professionals
   b) To compare the time required for designing dental prostheses for replacement of single missing tooth by AI and by dental professionals

<u>Research plan and methodology</u>

Part I (Objective 1)

Our AI system will **learn the relationship between individual teeth and rest of the dentition** using the 3D **Generative Adversarial Network (GAN)**.

The 3D models can be freely rotated in the virtual environment and the first step of learning the features of these 3D models is to align and orientate these teeth models to the occlusal plane. Segmentation of the 3D teeth model into individual teeth is not necessary in the GAN. There is several common methods to align and to interpret these 3D models for GAN (54) and the objective of this part of study is to investigate the best deep-learning algorithm for 3D teeth model.

Group 1: Voxel-based method

This method first partition the 3D model into regular cubes and then input into the neural network. Example of this algorithm includes VoxNet.

Group 2: View-based method (Multi-view)

This method uses multiple 2D image views of a 3D model for its alignment and orientation. Our group has expertise in performing image-to-geometry of the dental models. Our results showing that the use of anatomical landmarks at the occlusal surface e.g. cusp tips for aligning the 2D image to 3D model enable accurate image-to-geometry registration (33, 34). We will aggregates 2D image views from a loop around the 3D models and applies 2D deep learning framework to them. Example of this algorithm include Group-view convolutional neural networks (GVCNN).

Group 3: Point-based method (Point-cloud)

This method uses point-cloud to represent the 3D models and PointNet++ is an example. A point cloud is a set of data points in space. Each point has its set of X-, Y- and Z- coordinates. Point clouds are generally produced by 3D scanners and measure many points on the external surfaces of objects around them.

Group 4: Fusion method

This method learns on multiple types of data (multi-view, point-cloud and voxel) and fusion their features. Example of this algorithm include FusionNet uses the volumetric grid and multi-view for classification.

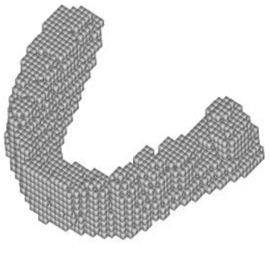

Voxel-based

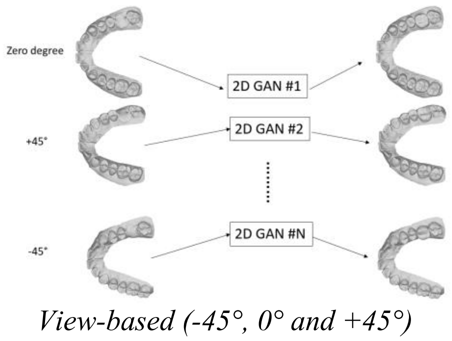

View-based (-45°, 0° and +45°)

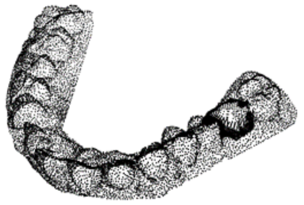

Point-based

We will collect 200 maxillary dentate teeth models as *training models*, and input them into our AI system. We will collect these models in the University teaching Hospital (Prince Philip Dental Hospital)

and the University dental clinics (University Health Services). Dentist will clinically examine the eligibility of the subject and the condition of individual teeth.

Selection criteria of subjects

Inclusion criteria:

- Subjects who have 12 or more occluding pair of natural teeth, from molar to molar
- Subjects who have stable intercuspal position (ICP) occlusion
- Subjects who have healthy periodontium

Exclusion criteria:

- Subjects who have received/are receiving orthodontic treatment
- Subjects who have periodontitis or history of periodontitis

Exclusion criteria of teeth:

- Teeth that have loss of nature morphology such as extensive decay, restoration or erosive tooth wear (cupping)
- Teeth that have pathological tooth movements such as fremitus, drifting, and overeruption

These excluded teeth will be removed from the model. After the initial training, the occlusal morphology and 3D position of the dental prostheses designed by AI system trained by these four groups will be compared with reference to the original natural teeth.

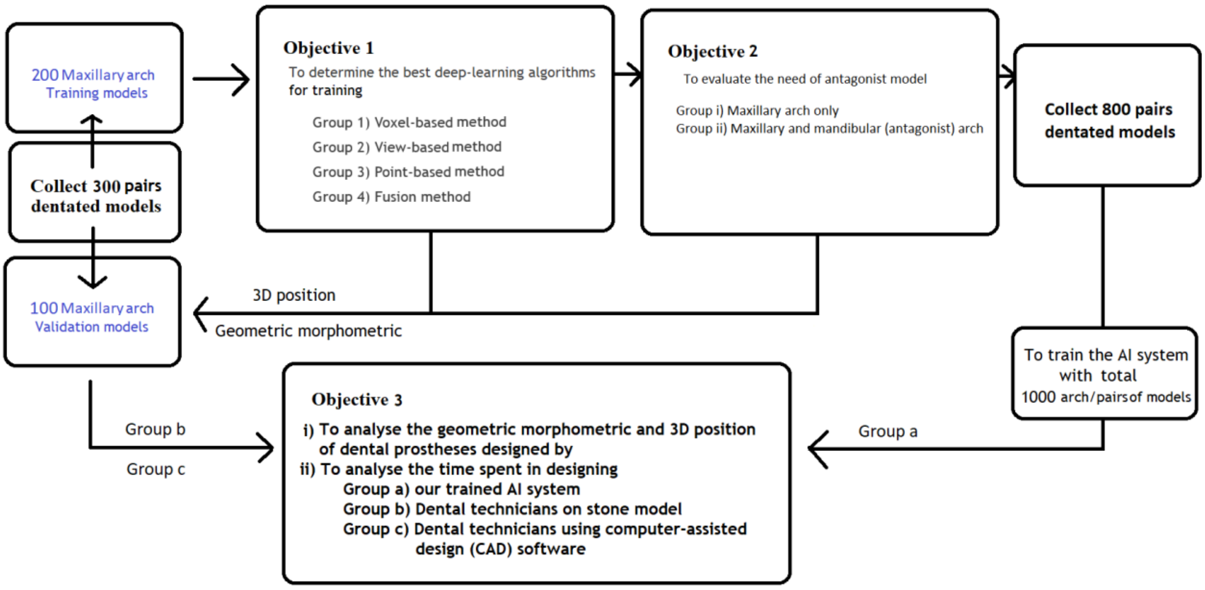

Part II (Objective 2)

After deciding the training algorithm in Part I, this part will investigate if the presence of antagonist (mandibular) teeth models should provide additional information to the AI system in predicting the occlusal morphology and 3D position of the dental prostheses. The antagonist teeth model will provide limitation to the occlusal surface and 3D position to the dental prostheses. There will be two groups **Group i) maxillary model only** and **Group ii) maxillary and mandibular (antagonist) model.**

The occlusal morphology and 3D position of the dental prostheses designed by AI system trained by these two groups will be compared with reference to the original natural teeth.

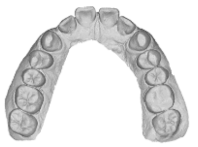

Maxillary arch only

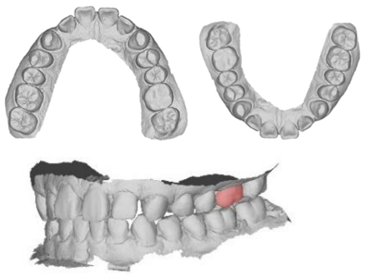

With antagonist model

Part III (Objective 3)

After deciding the training algorithm in Part I and the training data in Part II, this part will investigate if the Artificial Intelligence (AI) is better at predicting the occlusal morphology and 3D position of missing tooth/teeth based on the features of remaining dentition than conventional non-AI approaches. We will collect 800 more maxillary (+/- mandibular) dentate teeth models as training models, There will be three groups **Group a) our trained AI system; Group b) dental technicians on the physical models;** and **Group c) dental technicians assisted with CAD software**. We will investigate the time required for tooth design in these groups as secondary outcome.

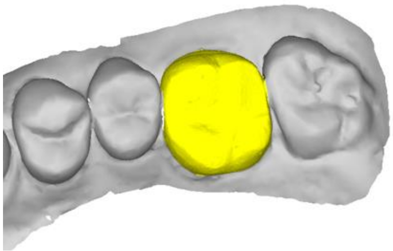

AI-generated

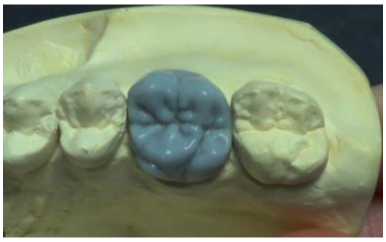

Dental technician on stone model

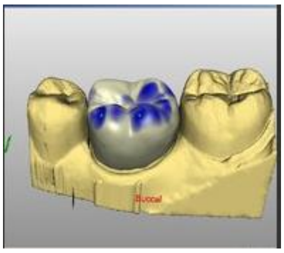

Dental technician using CAD software

Measurements

The occlusal morphology and 3D position of dental prostheses (experimental groups) will be compared to that of the healthy nature tooth (control).

Geometric morphometric of occlusal morphology

The dental prostheses will be superimposed to the original healthy teeth using best-fit algorithm. The **cusp tip and the center of the fossa/marginal ridge** (occlusal morphology) of the dental prostheses will be located and the difference in position to comparison to the control groups (distance in mm and direction) will be measured.

The experimental and control group will be superimposed to the adjacent teeth using best-fit algorithm. The **difference in the occlusal surface** between the dental prostheses and the natural teeth will be measured in mm^3 (Positive volume: **over-contoured**; Negative volume: **under-contoured**) and compared/analyzed as occlusal surface or as individual cusp.

Matching of tooth 16 to its arch

AI generated tooth 16 are used to match the trimmed tooth 16 to its corresponding arch.

3D position

The experimental and control group will be superimposed with reference to the adjacent teeth. The **center** of dental prostheses will be located and the difference to the center of natural teeth will be measured (distance in mm and direction) and compared.

Figure. Superimposition of teeth for comparison (Left). Measurement of the geometric morphology and 3D position by locating the anatomical landmarks of a tooth such as cusp tips and fossae as well as the center of a tooth (arrowed) respectively (Right)

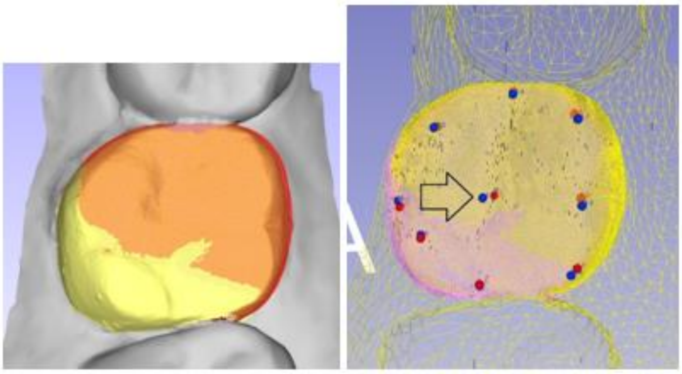

Time

The time (in minutes) spend in designing the dental prostheses by AI system and by dental technicians, with and without CAD software will be measured and analyzed.

Statistical and data analysis

One-way ANOVA (Part I and III) and t-test (Part II) will be used for comparison of experimental groups in this study. The result of different measurements within one part of study will be plotted into Receiver Operating Characteristic (ROC) curves.

Sample size calculation

The **recommended sample size for deep-learning in the medical imaging** 3D computed tomography has been found to be **1000** (55). For the determination of the best deep learning algorithm (objective 1) and if antagonist model is needed (objective 2), training with 200 models should be sufficient. From the below graphs, **the accuracy of AI system starts to reach plateau when the sample size reaches 200 and become saturated when reach 1000**. For Part I and II of this study, measurement will be made after input of 10, 20, 50, 100 and 200 teeth models. For Part III of this study, measurement will be made after input of 300, 500, 750 and 1000 (pair) teeth models.

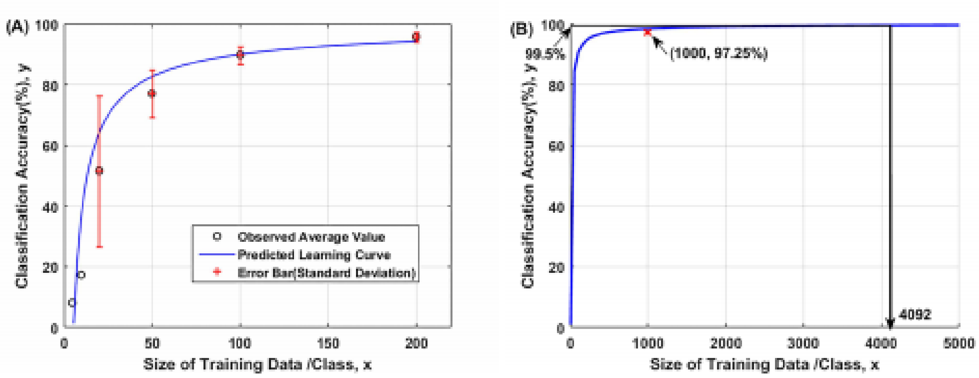

(A) The predicted learning curve and (B) tested result at large data set.

Adopted from "How much data is needed to train a medical image deep learning system to achieve necessary high accuracy?" (55).

Timeline

This is a 2-year clinical trial. The timeline and related manpower planning is attached.

Impact and significance

The strength of this study: 1) the use of healthy nature teeth for training of the AI system (instead of teeth build up by dental technicians); 2) there will be sufficient number of healthy nature teeth that are confirmed clinically for training; 3) this is one of the pioneering study in applying AI to deep-learn and predict 3D teeth model in dentistry.

Research

The scientific value of this project: 1) determine the deep learning algorithm for 3D teeth models; 2) determine the need of antagonist models for training; 3) prove the use of AI use in Prosthodontics.

This study will generate more research in the deep learning of the features of 3D models such as AI prediction of mandibular tooth, multiple missing teeth as well as clinical trials.

Education

This study will revolutionize the curriculum for training of dentists, dental technicians and associated professionals from human-driven to AI-driven and computer-driven approach.

References

1. Lin H, Corbet E, Lo E, Zhang H. Tooth loss, occluding pairs, and prosthetic status of Chinese adults. Journal of dental research. 2001;80(5):1491-5.
2. Department of Health HKSg. Oral Health Survey 2011.
3. Census and Statistics Department HKSg. Hong Kong Population Projections 2017-2066.
4. Geissler CA, Bates JF. The nutritional effects of tooth loss. The American journal of clinical nutrition. 1984;39(3):478-89.
5. McMillan AS, Wong M. Emotional effects of tooth loss in community-dwelling elderly people in Hong Kong. International Journal of Prosthodontics. 2004;17(2).
6. Fiske J, Davis DM, Leung KC, McMillan AS, Scott BJ. The emotional effects of tooth loss in partially dentate people attending prosthodontic clinics in dental schools in England, Scotland and Hong Kong: a preliminary investigation. International dental journal. 2001;51(6):457-62.
7. Wong M, McMillan AS. Tooth loss, denture wearing and oral health-related quality of life in elderly Chinese people. Community dental health. 2005;22(3):156-61.
8. Botelho MG, Ma X, Cheung GJK, Law RKS, Tai MTC, **Lam WYH**. Long-term clinical evaluation of 211 two-unit cantilevered resin-bonded fixed partial dentures. Journal of dentistry. 2014;42(7):778-84.
9. Botelho MG, Chan AW, Leung NC, **Lam WY**. Long-term evaluation of cantilevered versus fixed–fixed resin-bonded fixed partial dentures for missing maxillary incisors. Journal of Dentistry. 2016;45:59-66.
10. **Lam WY**, Botelho MG, McGrath CP. Longevity of implant crowns and 2-unit cantilevered resin-bonded bridges. Clinical oral implants research. 2013;24(12):1369-74.
11. Botelho MG, **Lam WY**. A fixed movable resin-bonded fixed dental prosthesis–A 16 years clinical report. Journal of prosthodontic research. 2016;60(1):63-7.
12. **Lam W**, Ho E. Rehabilitation of molar-incisor hypomineralization (MIH) complicated with localized tooth surface loss: a case report.
13. **Lam WY**, Chan RS, Li K, Tang K, Lui TT, Botelho MG. Ten-year clinical evaluation of posterior fixed-movable resin-bonded fixed partial dentures. Journal of dentistry. 2019;86:118-25.
14. Botelho MG, Dyson JE, Mui TH, **Lam WY**. Clinical audit of posterior three-unit fixed-movable resin-bonded fixed partial dentures–A retrospective, preliminary clinical investigation. Journal of dentistry. 2017;57:26-31.
15. **Lam WY**, McGrath CP, Botelho MG. Impact of complications of single tooth restorations on oral health-related quality of life. Clinical Oral Implants Research. 2014;25(1):67-73.
16. Magne P, Belser U. Bonded porcelain restorations in the anterior dentition: a biomimetic approach: Quintessence publishing company; 2002.
17. Magne P, Douglas WH. Rationalization of esthetic restorative dentistry based on biomimetics. Journal of Esthetic and Restorative Dentistry. 1999;11(1):5-15.
18. Posselt U. Physiology of occlusion and rehabilitation. 1962.
19. Christensen GJ. Is occlusion becoming more confusing?: A plea for simplicity. The Journal of the American Dental Association. 2004;135(6):770.

20. Piedra-Cascón W, Hsu VT, Revilla-León M. Facially driven digital diagnostic waxing: New software features to simulate and define restorative outcomes. Current Oral Health Reports. 2019;6(4):284-94.
21. Türp JC, Greene C, Strub J. Dental occlusion: a critical reflection on past, present and future concepts. Journal of oral rehabilitation. 2008;35(6):446-53.
22. Pan Y, Tam JM, Tsoi JK, **Lam WY**, Huang R, Chen Z, et al. Evaluation of laboratory scanner accuracy by a novel calibration block for complete-arch implant rehabilitation. Journal of Dentistry. 2020:103476.
23. Pan Y, Tam JM, Tsoi JK, **Lam WY**, Pow EH. Reproducibility of laboratory scanning of multiple implants in complete edentulous arch: effect of scan bodies. Journal of Dentistry. 2020:103329.
24. Molinero-Mourelle P, **Lam W**, Cascos-Sánchez R, Azevedo L, Gómez-Polo M. Photogrammetric and Intraoral Digital Impression Technique for the Rehabilitation of Multiple Unfavorably Positioned Dental Implants: A Clinical Report. Journal of Oral Implantology. 2019;45(5):398-402.
25. **Lam WY**, Luk HW, Ngan HY, Hsung RT, Goto TK, Pow EH. Validation of a Novel Geometric Coordination Registration using Manual and Semi-automatic Registration in Cone-beam Computed Tomogram. Electronic Imaging. 2016;2016(14):1-6.
26. **Lam WY**, Ngan HY, Wat PY, Luk HW, Goto TK, Pow EH, editors. Image calibration and registration in cone-beam computed tomogram for measuring the accuracy of computer-aided implant surgery. Image Processing: Machine Vision Applications VIII; 2015: International Society for Optics and Photonics.
27. **Lam WY**, Ngan HY, Wat PY, Luk HW, Pow EH, Goto TK, editors. Novel geometric coordination registration in cone-beam computed Tomogram. 2014 IEEE Applied Imagery Pattern Recognition Workshop (AIPR); 2014: IEEE.
28. Di Fiore A, Meneghello R, Graiff L, Savio G, Vigolo P, Monaco C, et al. Full arch digital scanning systems performances for implant-supported fixed dental prostheses: a comparative study of 8 intraoral scanners. Journal of prosthodontic research. 2019;63(4):396-403.
29. Alqahtani F. Marginal fit of all-ceramic crowns fabricated using two extraoral CAD/CAM systems in comparison with the conventional technique. Clinical, cosmetic and investigational dentistry. 2017;9:13.
30. Di AF, Vigolo P, Graiff L, Stellini E. Digital vs Conventional Workflow for Screw-Retained Single-Implant Crowns: A Comparison of Key Considerations. The International journal of prosthodontics. 2018;31(6):577-9.
31. **Lam WY**, Hsung RT, Choi WW, Luk HW, Pow EH. A 2-part facebow for CAD-CAM dentistry. The Journal of prosthetic dentistry. 2016;116(6):843-7.
32. **Lam WY**, Hsung RT, Choi WW, Luk HW, Cheng LY, Pow EH. A clinical technique for virtual articulator mounting with natural head position by using calibrated stereophotogrammetry. The Journal of prosthetic dentistry. 2018;119(6):902-8.
33. **Lam WY**, Hsung RT, Cheng LY, Pow EH. Mapping intraoral photographs on virtual teeth model. Journal of dentistry. 2018;79:107-10.

34. Hsung T-C, **Lam WY**, Pow EH, editors. Image to Geometry Registration for Virtual Dental Models. 2018 IEEE 23rd International Conference on Digital Signal Processing (DSP); 2018: IEEE.
35. Ender A, Mörmann WH, Mehl A. Efficiency of a mathematical model in generating CAD/CAM-partial crowns with natural tooth morphology. Clinical Oral Investigations. 2011;15(2):283-9.
36. Reich S, Trentzsch L, Gozdowski S, Krey K-F. In vitro analysis of laboratory-processed and CAD/CAM-generated occlusal onlay surfaces. International Journal of Prosthodontics. 2009;22(6).
37. Baroudi K, Ibraheem SN. Assessment of chair-side computer-aided design and computer-aided manufacturing restorations: a review of the literature. Journal of international oral health: JIOH. 2015;7(4):96.
38. Zhao Y, Wang Y. Understanding chair-side digital technology for stomatology from an engineering viewpoint. Zhonghua kou qiang yi xue za zhi= Zhonghua kouqiang yixue zazhi= Chinese journal of stomatology. 2018;53(4):230-5.
39. Güth J-F, Keul C, Stimmelmayr M, Beuer F, Edelhoff D. Accuracy of digital models obtained by direct and indirect data capturing. Clinical oral investigations. 2013;17(4):1201-8.
40. Akgungor G, Sen D, Bal E, Özcan M. Simultaneous replacement of maxillary central incisors with CEREC biogeneric reference technique: a case report. Journal of Dental Research, Dental Clinics, Dental Prospects. 2013;7(2):112.
41. Fiore AD, Monaco C, Brunello G, Granata S, Stellini E, Yilmaz B. Automatic Digital Design of the Occlusal Anatomy of Monolithic Zirconia Crowns Compared to Dental Technicians' Digital Waxing: A Controlled Clinical Trial. Journal of Prosthodontics.
42. Kurbad A, Kurbad S. Cerec Smile Design--a software tool for the enhancement of restorations in the esthetic zone. International journal of computerized dentistry. 2013;16(3):255.
43. Zhang R, Ding Q, Sun Y, Zhang L, Xie Q. Assessment of CAD-CAM zirconia crowns designed with 2 different methods: A self-controlled clinical trial. The Journal of prosthetic dentistry. 2018;120(5):686-92.
44. Miladinović M, Mihailović B, Mladenović D, Duka M, Živković D, Mladenović S, et al. Artificial intelligence in clinical medicine and dentistry. Vojnosanitetski pregled. 2017;74(3):267-72.
45. Li J, Shen Z, Xu WYT, **Lam WYH**, Hsung RTC, Pow EHN, et al. A compact dental robotic system using soft bracing technique. IEEE Robotics and Automation Letters. 2019;4(2):1271-8.
46. Li J, **Lam WYH**, Hsung RTC, Pow EHN, Wu C, Wang Z, editors. Control and Motion Scaling of A Compact Cable-driven Dental Robotic Manipulator. 2019 IEEE/ASME International Conference on Advanced Intelligent Mechatronics (AIM); 2019: IEEE.
47. LI J, Zhang Y, **Lam Y**, Hsung T, Pow E, Wang Z, editors. Robotic System for Automatic Dental Restorations in Oral Cavity. IEEE International Conference on Real-time Computing and Robotics (RCAR), 2018; 2018: IEEE.
48. Schwendicke F, Samek W, Krois J. Artificial Intelligence in Dentistry: Chances and Challenges. Journal of Dental Research. 2020:0022034520915714.

49. Grischke J, Johannsmeier L, Eich L, Griga L, Haddadin S. Dentronics: Towards robotics and artificial intelligence in dentistry. Dental Materials. 2020.
50. GH Li TH, WK Ling , **WY Lam**, G Pelekos. Automatic site-specific multiple level gum disease detection based on deep neural network. IEEE International Symposium on Biomedical Imaging (ISBI) (ISBI 2021) 2021.
51. Yuan F, Dai N, Tian S, Zhang B, Sun Y, Yu Q, et al. Personalized design technique for the dental occlusal surface based on conditional generative adversarial networks. International Journal for Numerical Methods in Biomedical Engineering. 2020;36(5):e3321.
52. Hwang J-J, Azernikov S, Efros AA, Yu SX. Learning beyond human expertise with generative models for dental restorations. arXiv preprint arXiv:180400064. 2018.
53. Bei M. Molecular genetics of tooth development. Current opinion in genetics & development. 2009;19(5):504-10.
54. Feng Y, Feng Y, You H, Zhao X, Gao Y, editors. MeshNet: Mesh neural network for 3D shape representation. Proceedings of the AAAI Conference on Artificial Intelligence; 2019.
55. Cho J, Lee K, Shin E, Choy G, Do S. How much data is needed to train a medical image deep learning system to achieve necessary high accuracy? arXiv preprint arXiv:151106348. 2015.
